# Revision of the Taiwanese millipede genus *Chamberlinius* Wang, 1956, with descriptions of two new species and a reclassification of the tribe Chamberlinini (Diplopoda, Polydesmida, Paradoxosomatidae, Paradoxosomatinae)

**DOI:** 10.3897/zookeys.98.1183

**Published:** 2011-05-13

**Authors:** Chao-Chun Chen, Sergei I. Golovatch, Hsueh-Wen Chang, Shyh-Hwang Chen

**Affiliations:** 1 Department of Biological Sciences, National Sun Yat-Sen University, 70 Lien-Hai Rd., Kaohsiung, Taiwan 804, ROC; 2 Institute for Problems of Ecology and Evolution, Russian Academy of Sciences, Leninsky pr. 33, Moscow 119071, Russia; 3 Department of Life Science, National Taiwan Normal University, 88 Tingchou Road, Sect. 4, Taipei, Taiwan 116, ROC

**Keywords:** Millipede, *Chamberlinius*, Chamberlinini, taxonomy, new species, key, distribution, Taiwan

## Abstract

The millipede genus *Chamberlinius* is basically confined to Taiwan, with only one of the four known species presumably introduced to southern Japan. Both previously known species are redescribed, based on new material: *Chamberlinius hualienensis* Wang, 1956 (the type species) and *Chamberlinius piceofasciatus* (Gressitt, 1941), the latter being a new subjective senior synonym of *Chamberlinius shengmui* Wang, 1957, **syn. n.** Two further congeners are described as new: *Chamberlinius pessior* **sp. n.** and *Chamberlinius sublaevus* **sp. n.** The genus is re-diagnosed, all of its four species are keyed, and their distributions mapped. The tribe Chamberlinini is reclassified and, based on gonopod traits, shown to comprise the following five genera: *Chamberlinius* Wang, 1956, *Haplogonosoma* Brölemann, 1916, *Riukiupeltis* Verhoeff, 1939, *Aponedyopus* Verhoeff, 1939 and *Geniculodesmus* Chen, Golovatch and Chang, 2008.

## Introduction

The genus *Chamberlinius* Wang, 1956 was first erected, based on the single species *Chamberlinius hualienensis* Wang, 1956, taken from Taruko, Hualien, Taiwan (Wang 1956). A year later, Wang (1957) described another new congener, *Chamberlinius shengmui* Wang, 1957, and he also proposed a new subfamily, Chamberlininae. Jeekel (1968) placed *Chamberlinius* in the tribe Sulciferini, subfamily Paradoxosomatinae. He compared the illustrations of *Orthomorphella pekuensis* (Karsch, 1881), the type species of *Orthomorphella* Hoffman, 1963, which Hoffman (1963) had validated, with the poor line drawings of both *Chamberlinius hualienensis* Wang, 1956 and *Chamberlinius shengmui* Wang, 1957 as presented by (Wang (1956, 1957), to conclude that *Chamberlinius* was a senior synonym of *Orthomorphella*. This opinion has since been shared by Wang and Mauriès (1996). Furthermore, Jeekel (1968) referred to *Chamberlinius* as containing four species: *Chamberlinius cristatus* (Takakuwa, 1942), *Chamberlinius hualienensis* Wang, 1956 (the type species), *Chamberlinius pekuensis* (Karsch, 1881) and *Chamberlinius shengmui* Wang, 1957.

Soon after that, Hoffman (1973), disagreeing with Jeekel (1968), resurrected *Orthomorphella* as an independent genus, removed *Chamberlinius* from the tribe Sulciferini, and downgraded the subfamily Chamberlininae to the tribal status, Chamberlinini in Paradoxosomatinae. This tribe was stated to encompass only *Chamberlinius* and *Riukiupeltis* Verhoeff, 1939. He was also the first to assign *Chamberlinius piceofasciatus* (Gressitt, 1941), another species from Taiwan, to *Chamberlinius*. He redescribed both *Chamberlinius hualienensis* and *Chamberlinius piceofasciatus*,simply listing *Chamberlinius shengmui* as a third congener. More recently, Hoffman (1980) spoke already of “about five species” in this genus, which is a slightly puzzling estimate because only the above trio has hitherto been known to compose *Chamberlinius*.

Regrettably, since all of the diplopod material (Wang (1956, 1957) claimed to have deposited in the Department of Zoology (now incorporated into the Department of Life Science) of the National Taiwan University and/or Taiwan Museum seems to be lost, it appears necessary to amass new samples, including topotypes, and build up a modern collection of Taiwanese Diplopoda.

The present study is a revision of the millipede genus *Chamberlinius*, based not only on new material, including some topotypes, but also on a few old types. As a result, all previously described species could be re-assessed, two new species added, and a new synonym established. In addition, the tribe Chamberlinini could be reclassified due to the present revision of the type genus *Chamberlinius*.

In his Nomenclator, Jeekel (1971, p. 219) corrected Chamberlininae to Chamberliniinae, because family-group names are derived from the stem of the genitive singular of the genus name, which in this case is Chamberlinii, stem Chamberlini-. Similarly, the correct form of Chamberlinini is Chamberliniini. We are keeping Chamberlininae and Chamberlinini because Jeekel’s correction was not widely adopted and these names are in prevailing usage (ICZN section 29.5).

## Material and methods

New extensive collections of millipedes covering most parts of Taiwan were made between 1986 and 2010, using hand-sorting of the soil and litter. Specimens were preserved in 70% ethanol. External morphology was examined and the drawings prepared with a LEICA MZ 16 stereomicroscope as well as with a HITACHI S2400 scanning electron microscope. Coloration of the specimens is described from alcohol material. This material has been shared between the collections of Department of Life Sciences, National Chung-Hsing University (NCHUL), Taiwan; National Museum of Natural Science (NMNS), Taiwan; the Department of Biological Sciences, National Sun Yat-Sen University (NSYSUB), Taiwan; Department of Life Science, National Taiwan Normal University (NTNUL), Taiwan; Taiwan Forestry Research Institute (TFRI), Taiwan; California Academy of Sciences (CAS), San Francisco, U.S.A.; National Museum of Natural History (NMNH), Smithsonian Institution, Washington, D.C., U.S.A.; Field Museum of Natural History (FMNH), Chicago, Illinois, USA, and Zoological Museum of the State University of Moscow (ZMUM), Russia.

## Systematic account

### Genus Chamberlinius

Wang, 1956

http://species-id.net/wiki/Chamberlinius

Chamberlinius Wang 1956: 156, 1957: 103; Jeekel 1968: 73; Jeekel 1971: 219; Hoffman 1973: 377, 1980: 170; Korsós 2004: 22.

#### Diagnosis

 (amended): Large-sized Chamberlinini (25–37 mm long).Paraterga very well-developed, paraterga 2 not lower, but as high as adjacent paraterga. Pleurosternal carinae well-developed. Both a lobe between ♂ coxae 4 and adenostyles missing. A pair of ridge-like spiracles flanking gonopod aperture.

Gonopod coxae long, subcylindrical, setose distodorsally, cannula as usual. Telopodites very slender and long, in situ their distal parts crossing medially. Femorite especially long, longer than acropodite, simple, devoid of outgrowths, apically with a clear cingulum demarcating a mesally directed, elongate postfemoral part; the latter branching near base (just distal to cingulum) into a long, thick and simple solenomere and a shorter to longer, similarly thick solenophore supplied with a dentiform outgrowth at base. Seminal groove running mostly on medial face of femorite to move laterally only towards, and to return back to medial side near, cingulum.

#### Type species:

*Chamberlinius hualienensis* Wang, 1956, by original designation.

#### Remarks.

Hoffman (1973), when outlining the tribe Chamberlinini, included only two genera therein: *Chamberlinius* and *Riukiupeltis* Verhoeff, 1939, with three and one species, respectively. They show very well-developed paraterga resembling those of some Xystodesmidae, both a lobe between ♂ coxae 4 and adenostyles missing, and the gonopods being peculiar in the entire terminal half of the telopodite (distal to the cingulum) representing a single element with several subterminal branches, one of which carries the seminal groove.

Such a description of gonopod structure actually means that, unlike many other tribes of Paradoxosomatidae, which have a long and flagelliform solenomere more or less strongly sheathed/protected by a complex, rather membranous solenophore, the solenomere in the Chamberlinini is a thick, strong and long branch accompanied by a similarly thick, strong and long solenophore branch. The above new diagnosis of *Chamberlinius* emphasizes its peculiar gonopod conformation. Regrettably, the gonopod conformation of *Riukiupeltis* still remains to be clarified to properly rediagnose this genus (Jeekel 1968).

Jeekel (1968) included the following species in *Chamberlinius*: *Chamberlinius cristatus* (Takakuwa, 1942) from Japan, *Chamberlinius pekuensis* (Karsch, 1881) from Japan, Korea, continental China and Taiwan, *Chamberlinius hualienensis* (misspelled as *hauliensis*) Wang, 1956 and *Chamberlinius shengmui* Wang, 1957, both latter taxa from Taiwan. Hoffman (1973) later transferred *pekuensis* into *Orthomorphella* (see above). As regards *Chamberlinius cristatus*, originally described as *Orthomorpha cristatus* Takakuwa, 1942 from near Tokyo, Japan (Takakuwa 1942), this species badly needs a revision (Jeekel 1968), but the sketch of its gonopod structure alone, showing a flagelliform solenomere and a complex membranous solenophore, prevents its assignment to Chamberlinini altogether. So *Orthomorpha cristatus* is here formally ejected from *Chamberlinius*, but its current generic relationships remain obscure.

As a result, presently only three species can be considered as belonging to this genus (Hoffman 1973, Korsós 2004). Our contribution puts on record two new congeners from Taiwan and establishes the synonymy of one of the older species, altogether providing a revision of *Chamberlinius*.

#### Distribution:

 All *Chamberlinius* species are believed to be native to Taiwan (Hoffman 1980), but one congener seems to have been introduced to Japan (Higa and Kishimoto 1986, 1989, Yamaguchi et al. 2000, Niijima and Arimura 2002, Nakamura and Korsós 2010).

#### Chamberlinius hualienensis

Wang, 1956

http://species-id.net/wiki/Chamberlinius_hualienensis

Figs 1–8 33–35

Chamberlinius hualienensis Wang 1956: 155, fig. 1, 1958a: 341, 1958b: 881, 1963: 90, 1964: 69; Jeekel 1968: 70, 1971: 219; Hoffman 1973: 379, figs 19–21; Higa and Kishimoto 1986: 62–72, figs 3, 9–14; Wang and Mauriès 1996: 87; Korsós 2004: 32.

##### Material examined:

 1 ♀ (NSYSUB-DI 38), Taipei City (台北市), Yangmingshan National Park (陽明山國家公園), Rd. BaLaKa (巴拉卡公路), under decayed leaves in a gutter, ca. 850 m a.s.l., 10 September 2002, leg. S. Y. Wu. 1 ♂ (NSYSUB), same locality, the first staff dormitory, 24 June 2003, leg. J. L.Chao. 1 ♂ (NSYSUB-DI 01), New Taipei City (新北市), Wulai District (烏來區) (formerly: Taipei County 台北縣, Wulai Township 烏來鄉), Wulai (烏來), 5 November 2001, same collector. 1 ♀ (NSYSUB-DI 07), same place, Neidong (內洞), ca. 500 m a.s.l., 16 August 2002, leg. C. C. Chen. 1 ♀ (NSYSUB-DI 06), Tauyuan County (桃園縣), Fushing Township (復興鄉), Hualing (華稜), 1,060 m a. s. l., 19 August 2002, leg. J. N. Huang. 2 ♂, 2 ♀ (NSYSU), Taichung City (台中市), Hoping District (和平區) (formerly: Taichung County 台中縣, Heping Township 和平鄉), Anmashan Forest Rd. (鞍馬山林道), 2,000–2,500m a.s.l., 1 June 2003, leg. J. D. Lee. 2 ♂, 1 ♀ (NSYSU), same district, Basianshan Forest Recreation Area (八仙山森林遊樂區), 900–1,500 m a.s.l., 23 June 2010, leg. H. D. Zhu. 1 ♂ (NCHUL), Nantou County (南投縣), Yuchi Township (魚池鄉), Lake Sun Moon (日月潭), ca. 750 m a.s.l., 16 July 2004, leg. S. H. Wu. 1 ♂, 1 ♀, same county, Lugu Township (鹿谷鄉), Siitou (溪頭), ca. 1,200 m a. s. l., 15 November 2002, leg. J. D. Lee. 4 ♂, 3 ♀ (NSYSU), same county, Xinyi Township (信義鄉), Tongfu Village (同富村), Provincial Highway # 21 (another name: New Central Cross-Island Highway 新中橫公路), in soil, 1,280 m a.s.l., 23°34'09"N, 120°53'29"E, 8 September 2004, leg. H. D. Zhu. 3 ♂, 2 ♀, 1 juvenile (NSYSUB), same township, Dongpu (東埔), Shalisian Stream (沙里仙溪), in soil under stones, ca. 1,020 m a.s.l., 23°33'24"N, 120°55'19"E, 9 April 2004, same collector. 2 ♂, 1 ♀ (NSYSU), same county, Zhushan Township (竹山鎮), Shanlinsi Stream (杉林溪), ca. 1,600–2,000 m a.s.l., 7 October 2004, leg. S. Y. Wu. 1 ♂, 2 juveniles (NCHUL), Chia-i County (嘉義縣), Alishan Township (阿里山鄉), 65 road-km of Provincial Highway # 18, near Shihjhuo (石卓), ca. 1,350 m a.s.l., 28 October 2000, leg. H. Z. Liang. 1 ♂, 1 ♀, 1 ♂ juvenile (NSYSUB-DI 02-04), same county, Fanlu Township (番路鄉), Longtou (巃頭), in bamboo forest, ca. 1,300 m a.s.l., 21 April 2002, leg. J. L.Chao. 1 ♂ (NTNUL-My 70), Kaohsiung City (高雄市), Maolin District (茂林區) (formerly: Kaohsiung County 高雄縣, Maolin Township 茂林鄉), Shanping (扇平), ca. 700 m a.s.l., 7–9 July 1986, leg. S. H. Chen. 3 ♂, 6 ♀ (NTNUL-My 16–24), 26 January 1989, same place and collector. 1 ♀ (NSYSUB-DI 05), same place, 13 May 2002, leg. C. H. Yang. 1 ♀ (NSYSUB), same place, 1 October 2006, leg. M. H. Hsu. 1 ♂ (NSYSU), same district, Duona Township Rd. (多納林道), 1,600 m a.s.l., 11 August 2004, leg. T. Y. Tei. 1 ♀ (NSYSUB-DI 58), same city, Taoyuan District (桃源區) (formerly: Taoyuan Township 桃源鄉), Tengjhih (藤枝), ca. 1,450 m a.s.l., 21 October 2001, leg. S. Y. Wu. 1 ♂, 2 ♀ (NSYSUB-DI 35–37), Pingtung County (屏東縣), Shihzih Township (獅子鄉), Neiwun (內文村), in humus under decayed wood, ca. 400–500 m a.s.l., 5 October 2002, same collector. 5 ♂, 3 ♀, 6 juveniles (NSYSU), same county, Mudan Township (牡丹鄉), Dongyuan (東源), about 300 a.s.l., 4 July 2006, leg. H. W. Chang. 1 ♀ (NSYSU), Ilan County (宜蘭縣), Jiaoxi Township (礁溪鄉), Ilan village street (宜6鄉道), Linmeishipan forest path (林美石盤林道), 200 m a.s.l., 8 September 2009, leg. C. C. Cheng. 9 ♂, 3 ♀ (NSYSUB-DI 18–29), same county, Datong Township (大同鄉), Ciilan Forest Amusement Park (棲蘭森林遊樂園), 460–480 m a.s.l., 20 August 2002, leg. C. C. Chen and Y.H. Lin. 2 ♂, 5 ♀ (NSYSU), same township, Renze Warning Spring (仁澤溫泉), 560 m a.s.l., 24°32'42"N, 121°30'16"E, 29 August 2004, leg. H. D. Zhu. 1 ♂, 4 juveniles (NSYSUB-DI 30-34), same township, Taipingshan Working Station (太平山工作站), under decayed leaves, ca. 480 m a.s.l., 20 August 2002, leg. C. C. Chen, Y. H. Lin, J. N. Huang etc. 8 ♂, 2 ♀ (NSYSUB-DI 08-17), same township, Sihji Forest Rd. (四季林道), ca. 1,060 m a.s.l., under fallen bamboo leaves, 20 August 2002, leg. C. C. Chen and Y.H. Lin. 2 ♂, 1 ♀ (ZMUM), 2 ♂, 1 ♀ (FMNH 6673-6675), 2 ♂, 1 ♀ (NMNS 4418-001), same place, date and collectors. 1 ♂, 1 ♀ (NSYSU), same township, Sihji Village (四季村), ca. 780 m a.s.l., 24°29'32"N, 121°25'43"E, 9 April 2006, leg. M. H. Hsu. 1 ♂ (NSYSU), same township, Nanshan Village (南山村), ca. 1,380 m a.s.l., 24°27'08"N, 121°22'51"E, 8 April 2006, same collector. 3 ♂, 2 ♀ (NSYSU), Hualien County (花蓮縣), Xiulin Township (秀林鄉), Tongmen Village (銅門村), Provincial Highway # 14 (台14線), ca. 210 m a.s.l., 23°58'39"N, 121°28'28"E, 13 February 2007, same collector. 1 ♀ (NSYSU), same county, Xincheng Township (新城鄉), Beipu Village (北埔村), productive road (產業道路), ca. 80 m a.s.l., 24°03'22"N, 121°35'35"E, 1 March 2007, same collector. 2 juveniles (NSYSU), same county, Fengbin Township (豐濱鄉), Hualien village street 51 (花51鄉道) (Baliwan Productive Road 八里灣產業道路), ca. 250 m a.s.l., 23°35'15"N, 121°30'15"E, 6 May 2009, same collector. 16 juveniles (NSYSU), same place, 23°35'06"N, 121°30'04"E, same date and collector. 3 juveniles (NSYSU), same county, Rueisuei Township (瑞穗鄉), Rueigang Highway (瑞港公路), Houzihshan (猴子山), ca. 130 m a.s.l., 23°29'50"N, 121°25'23"E, 7 May 2009, same collector. 2 juveniles (NSYSU), Taitung County (台東縣), Changbin Township (長濱鄉), Sanjianwn (三間屋), ca. 150 m a.s.l., 23°21'37"N, 121°27'33"E, 7 May 2009, same collector. 2 ♂, 3 ♀ (NSYSU), same county, Haiduan Township (海瑞鄉), Xiangyang (向陽), mine, under stones mixed with fallen leaves, ca. 1,280 m a.s.l., 26 June 2003, leg. S. Y. Wu. 2 ♂ (NSYSU), same township, South Cross-island Highway 175 k (near Lidao 利稻), ca. 1,070 m, a.s.l., 26 June 2003, same collector. 1 ♀ (NSYSU), same township, tunnel entrance of Wulu (霧鹿隧道口), in fallen leaves in a gutter, ca. 710 m a.s.l., 26 June 2003, same collector. 1 ♂ (NSYSU), same county, Beinan Township (卑南鄉), Lijia Forest Rd. (利嘉林道, also called Lijia Productive Road 利嘉產業道路), ca. 1,340 m a.s.l., 22°49'56"N, 121°00'34"E, 18 August 2004, leg. Y. J. Fan. 9 juveniles (NSYSU), same township, Yanping Forest Rd. (延平林道), ca. 1,070 m a.s.l., 22°52'58"N, 121°02'18"E, 3 June 2009, leg. M. H. Hsu. 1 ♂, 1 ♀ (NSYSU), same place, 22 February 2007, leg. S. Y. Wu. 1 ♂, 1 juvenile (NSYSU), same county, Taimali Township (太麻里鄉), Yima forest path (依麻林道), 6 December 2004, < 800 m a. s. l., same collector. 1 ♀ (NSYSU), same county, Jinfeng Township (金峰鄉),Forestry Research Institute Forest Rd. (林試分所林道), ca. 850 m a.s.l., 30 August 2003, leg. M. H. Hsu.

##### Description:

**Figures 1–8.**
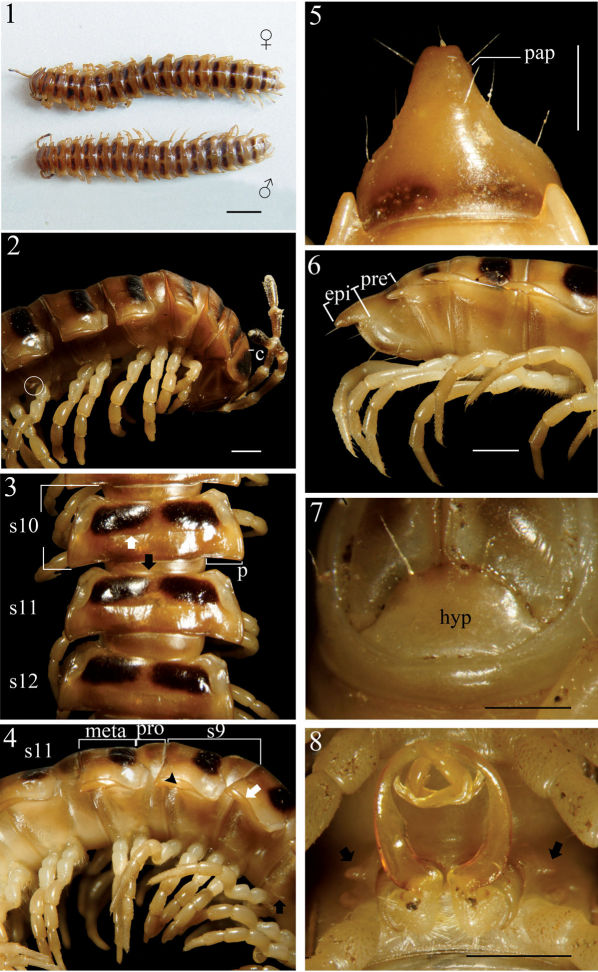
*Chamberlinius hualienensis* Wang, 1956, ♂ and ♀ from Siji forest path (四季林道) (1) and ♂ from Basianshan Forest Recreation Area (八仙山森林遊樂區) (2–8). **1** Entire body, dorsal view **2** Anterior body portion, lateral view. Circle: caudal tooth of pleurosternal carinae **3** Segments 10–12, dorsal view. up arrow: transverse sulcus; down arrow: stricture **4** Segments 9–11, lateral view. down arrow: calluses; up arrow: pleurosternal carinae; triangle: ozopore **5, 6** Epiproct, dorsal and lateral views, respectively **7** Hypoproct, ventral view **8** Spiracle-bearing cones lateral to gonopod aperture (arrows). Scale bars: 5.0 mm (1); 1.0 mm (2–6, 8); 0.5 mm (7). **c**:collum; **epi**: epiproct; **hyp**: hypoproct; **meta**: metatergum; **p**:paraterga; **pap**: pre-apical papillae; **pro**: prozona; **s9-s12**: segments 9–12 separately.

Length 30–34 (♂, n=5) and 32–37 mm (♀, n = 5); width of midbody metaterga 10 ca. 4.0–5.0 (♂) and 5.0–5.2 mm (♀). Coloration of both sexes in alcohol (Fig. 1) almost uniformly very light brown to brown in both sexes; head, collum (except posterior edge), anterior end of metaterga 2–4, sometimes also of 19th, brown, subtrapeziform (Fig. 3) on presulcus halves of metaterga 5–18, separated by an axial line, closer to axial line with narrower sides but closer to paraterga with wider sides; antennae entirely light brown to increasingly blackish distally; tip contrastingly pallid.

In width, head < collum (**c**) (Fig. 2) = segment 2 > 3 < 4 < 5 < 6–9 < 10 < 11 < 12 < 13 < 14 < 15 ≥ 16 in ♂, but head < collum = segment 2 > 3 < 4 < 5 < 6–9 < 10 < 11 < 12 < 13 < 14 in ♀; thereafter body gradually and gently tapering both in width and height towards telson. Antennae medium-sized to long, slender, reaching behind middle to posterior end of metatergum 4 (Fig. 2) dorsally in ♂, or posterior end of segment 3 to anterior edge of segment 4 in ♀. Surface generally shining and smooth, rugulose on metazona (Figs 3, 4), also evidently and densely granular below paraterga 2–19 in both sexes. Paraterga (**p**) (Fig. 3) very well-developed, sometimes slightly more strongly in ♂ as compared to ♀, calluses (Fig. 4, down arrow) delimited by a sulcus dorsally and ventrally on segments 2–19, ventral sulcus finer than dorsal one; paraterga like high ridges, subhorizontal, extending increasingly beyond caudal tergal margin on segments 2–19 in both sexes, especially strongly spiniform caudally on segments 17–19 (♂) (Fig. 5) or 18 and 19 (♀); anterior corner of paraterga thinner dorsoventrally and depressed much more obviously in ♂ (Fig. 4). Axial line (Fig. 3) absent or traceable in places, usually present. Transverse sulcus (Fig. 3, up arrow) evident on metaterga 5–17, poorly traceable to evident on metatergum 18, wanting on segment 19 in both sexes, narrow, shallow, slightly deeper in ♂, neither beaded at bottom nor reaching bases of paraterga in both sexes. Limbus thin, caudal margin entire. Segments poorly constricted. Stricture (Fig. 3, bottom) dividing pro- and metazona (Figs 3, 4) shallow, moderately wide, densely and roughly beaded at bottom dorsally down to paratergal level, contrastingly more roughly so in ♀ as compared to ♂. Pleurosternal carinae (Figs 2, 4) well-developed, contrastingly more strongly so in ♂ as compared to ♀, well-developed on segments 2–9(10), traceable on segments 10–14, like low bosses on segments 15 and 16 to sometimes only traceable on segments 11–17 in ♂, or well-developed on segments 2–10, traceable on segments 11 and 12, like low bosses on segments 13–15 to sometimes only traceable on segments 11–16 in ♀, with small caudal teeth on segments 3–7(8) (♂) (Fig. 2, left) or (2)3–8 (♀), thereafter wanting. Tergal setae fully abraded, pattern untraceable in both sexes. Ozopores (Fig. 4, triangle) lateral, lying on callus ca. one-third metatergal length in front of caudal edge. Epiproct (**epi**) (Figs 5, 6) flattened dorsoventrally, long, somewhat longer in ♂ as compared to ♀, curved (especially well so in ♂) and directed caudoventrad in lateral view (Fig. 6), ratio of epiproct length to pre-epiproct (**pre**) length of telson 1:1.8 in ♂ (Fig. 6), tip subtruncate to slightly concave (♂) or emarginate (♀) in dorsal view (Fig. 5); pre-apical papillae (**pap**) evident (Fig. 5), situated close to apex. Hypoproct (**hyp**) (Fig. 7) subtrapeziform, caudally rounded (♂) or convex to rounded (♀), 1+1 setae at caudal corners situated on well-separated knobs, sides slightly concave at base in both sexes.

Sterna moderately setose in ♂, more sparsely so in ♀; an obvious, short, round ridge supporting spiracles lateral to gonopod aperture (Fig. 8, arrows); each cross-impression with an evident transverse sulcus, but without axial groove. Legs (Fig. 4) without tarsal brushes, long, ca. 1.5 times as long as midbody height in ♂, slightly shorter in ♀. Each ♂ coxa 2 perforated by a vas deferens with a very small pore opening apically.

Gonopods (Figs 33–35) simple; coxae (**co**) long, subcylindrical, setose distodorsally; prefemur (**prf**) large, short, almost 1/4 femur length, densely setose; femorite (**f**) about midway evidently thinner and broadened, with a thin membranous lobe on mesal side (**l**); cingulum marking the end of femorite very clear, expecially so on lateral side, common stem of postfemoral region (**pof**) short, very quickly branching into a remarkably long and strong solenomere (**sl**) crowned with a narrow apical lamina (**m**), and a similarly long, simple, less strong and unequally bifid solenophore (**sph**) with a short, strong, dentiform process (**dp**).

##### Distribution:

*Chamberlinius hualienensis* Wang, 1956 is currently known from Taiwan, as well as on Kyushu Island and on most of the islands of the Ryukyu Archipelago, Japan (Higa and Kishimoto 1986, 1989, Yamaguchi et al. 2000, Niijima and Arimura 2002, Nakamura and Korsós 2010). The occurrences of C.*hualienensis* in Japan are likely to be introduced from Taiwan through human agency. Moreover, it is this species in Japan, but not in Taiwan, that occasionally shows swarming, like repeated massive outbreaks in Okinawa (Higa and Kishimoto 1986) or one at Kagoshima in early winter 2000 which caused serious railway traffic problems (Niijima and Arimura 2002).

Such a distribution pattern vividly reminds of that demonstrated by still another basically Taiwanese paradoxosomatid genus, *Aponedyopus* Verhoeff, 1939. Of its three currently known species (Chen et al. 2010), only one, the most widespead *Aponedyopus montanus* Verhoeff, 1939, has been recorded in the Ryukyus, Japan, but its identity still requires verification (Nakamura and Korsós 2010).

In Taiwan, *Chamberlinius hualienensis* is likewise the most common and widespread among its congeners, with altitudes ranging from about 80 m to ca. 2,500 m a.s.l. (Map).

**Map.**
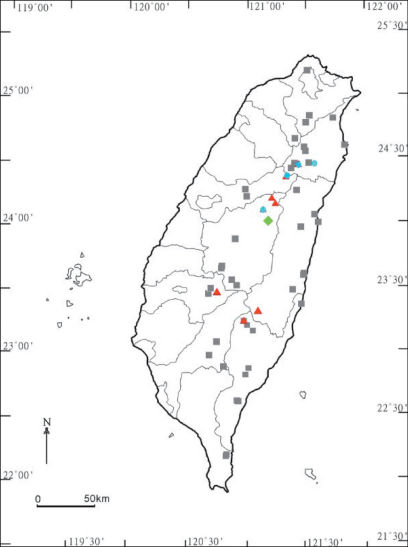
Distribution of *Chamberlinius* species in Taiwan. *Chamberlinius hualienensis* Wang, 1956: filled grey squares; *Chamberlinius piceofasciatus* (Gressitt, 1941): filled red triangles; *Chamberlinius pessior*sp. n.: filled light-green diamond. *Chamberlinius sublaevus* sp. n.: filled blue circles.

#### Chamberlinius piceofasciatus

(Gressitt, 1941)

http://species-id.net/wiki/Chamberlinius_piceofasciatus

Figs 9–16 39–41 45, 46 53

Prionopeltis piceofasciatus Gressitt 1941: 59; Wang 1964: 69; Jeekel 1968: 69.
Chamberlinius piceofasciatus Hoffman 1973: 381, figs 23–27; Wang and Mauriès 1996: 87; Korsós 2004: 32.
Chamberlinius shengmui Wang 1957b: 103, fig. 4; Hoffman 1973: 382; Korsós 2004: 22. **New Synonymy!**

##### Material examined:

 1 ♂, 1 ♀ (CAS, type series number # 5617 of *Prionopeltis piceofasciatus*: contrary to Hoffman’s (1973) presentation, it is the female that Gressitt (1941) had labeled as the holotype, while the male is a paratype), central Taiwan (臺灣中部), Arisan (阿里山), 2,000 m a.s.l., 24 May, 1934, leg. J. L. Gressitt. 1 ♂, 1 ♀ (NMNH, *Chamberlinius shengmui*, det. & ded. Y. H. M. Wang), Alisan Kiayi (阿里山，嘉義縣), August, 1957, leg. Y. H. M. Wang. 1 ♀ (TFRI), Taichung City (台中市), Hoping District (和平區) (formerly: Taichung County 台中縣, Heping Township 和平鄉), Shengguang (勝光), 2,000–2,300 m a.s.l., 21 August - 24 September 2002, leg. W. C. Yeh. 1 ♂ (TFRI), same place, date and collector. 1 ♀ (TFRI), same place, 24 November - 24 December 2002, same collector. 1 ♂ (TFRI), same place, 26 March - 25 April 2003, same collector. 1 ♂ (TFRI), same place, 24 July 2003, same collector. 1 ♂ (TFRI), same place, date and collector. 1 ♀ (NTNUL-My 42), Nantou County (南投縣), Renai Tow nship (仁愛鄉), Hehuanshan (合歡山), 3,100 m a.s.l., 30 August 1988, leg S. H. Chen. 1 ♂ (NCHUL), same township, Meifeng (梅峰), ca. 2,000 m a.s.l., 2 April 2002, leg. S. H. Wu. 1 ♂, 2 ♀, 3 juveniles (NSYSUB-DI 44-49), same township, HuaGer water source (華岡水源), ca. 2,600 m a.s.l., 22 August 2002, leg. C. C. Chen and J. N. Huang. 3 ♂, 2 ♀ (NTNUL-My 1-5), Chia-I County (嘉義縣), AliShan (阿里山), 2,260 m a.s.l., 3 July 1989, leg. S. H. Chen. 1 ♂, 1 ♀ (NTNUL-My 59-60), same place, 2,250 m a.s.l., same date and collector. 1 ♂ (TFRI), Ilan County (宜蘭縣), Datong Township (大同鄉), Lakes Jialuohu (加羅湖), ca. 2,300 m a.s.l., 24 May 2002, leg. W. C. Yeh. 1 juvenile (TFRI), same place, 23 August 2002, same collector. 1 ♂ (NCHUL), Hualien County (花蓮縣), Zhuoxi (卓溪), 2,500 m a.s.l., 15 February 2008, leg. S. H. Wu. 1 ♂ (NTNUL-My 43), Taitung County (台東縣), Haituan Township (海端鄉), Siangyang (向陽), on wall, ca. 2,270 m a.s.l., 3 September 2002, leg. J. H. Chen.

##### Diagnosis:

 Closest to C. *hualienensis*, but differs in often a lighter general coloration; in metaterga 2–19 with only a slightly infuscate (brown), subtrapeziform band in the anterior half (versus 1+1 darker, axially separated spots in *Chamberlinius hualienensis*); in the paraterga like low ridges (versus higher ridges in *Chamberlinius hualienensis*); by the pleurosternal carinae with small caudal teeth on segments 3–5(9, 10) (♂) (versus 3–7(8) in *Chamberlinius hualienensis*); in the epiproct shorter; in the smaller spiracle-bearing ridges lateral to the gonopod aperture; and in the gonopods, in which the solenophore is rounded apically and considerably shorter than the solenomere, while the parabasal dentiform process is stouter and more solid (versus longer and membranous in *Chamberlinius hualienensis*), always placed behind the solenomere in ventral view.

##### Description:

 Length 29–33 (♂, n= 4) or 31–34 mm (♀, n= 3); width of metazonite 10 ca. 4–4.5 (♂) or 4.5–5.0 mm (♀). Specimens in NMNH: Length ca. 32 (♂, n=1) and 38 mm (♀, n = 1**)**; width of midbody metaterga 10 ca. 4.0 (♂) and 4.3–4.5 mm (♀).

**Figures 9–16.**
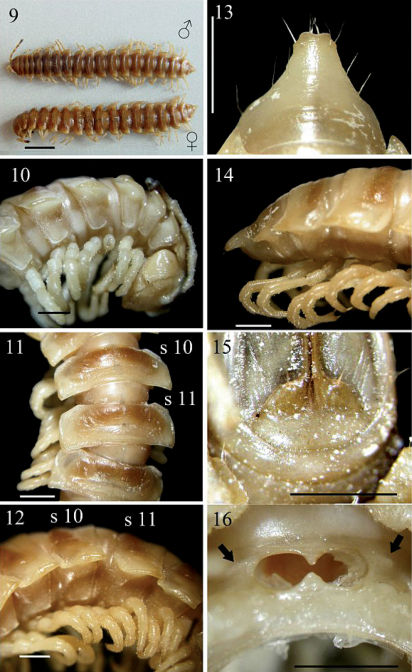
*Chamberlinius piceofasciatus* (Gressitt, 1941), ♂ and ♀ from HuaGer water source (華岡水源) (9) and ♂ from AliShan (阿里山) (10**–**16). **9** Entire body, dorsal view **10** Anterior body portion, lateral **11, 12** segments 10 and 11, dorsal and lateral views, respectively **13, 14** Epiproct, dorsal and lateral views, respectively **15** Hypoproct, ventral view **16** Spiracle-bearing cones/ridges lateral to gonopod aperture (arrows). Scale bars: 5.0 mm (9); 1.0 mm (10–16). **s10** and **s11**: segments 10 and 11 separately.

Coloration in alcohol (Fig. 9) light yellow-brown to light brown from head to end of epiproct, as well as from dorsum down to paraterga, sterna and legs; collum and metaterga 2–19 with a slightly infuscate (brown), subtrapeziform band in anterior half (Fig. 11); a lighter or darker anterior part of epiproct; colour pattern similar in both sexes, but ♀ darker; antennae increasingly blackish distally, but tip contrastingly pallid.

In width, head < collum < 2 > 3 < 4 < 5 < 6 < 7 < 8–16 in ♂, or head < collum ≤ 2 > 3 < 4 < 5 < 6 < 7 < 8 < 9–16 in ♀; thereafter body gradually and gently tapering both in width and height towards telson. Antennae (Figs 9, 10) moderately long to long in ♂, slender, reaching either behind posterior end of metatergum 4 to anterior end of metatergum 5 (♂), or anterior edge to end of metatergum 3 (♀) dorsally. Surface smooth throughout, rugulose on metaterga (Figs 10, 12) and below paraterga where evidently and densely granular on segments 2–19 in ♂; sometimes so only on segments 2–5, suddenly not so densely on segments 6 and 7, then wanting on segments 8–14, but traceable again on segments 15 and 16 in ♂ or on segments 2–18 in ♀. Paraterga (Fig. 11) very well-developed, calluses (Fig. 12) delimited by a sulcus only dorsally on segments 3 and 4, both dorsally and ventrally on segments 5–19; like low ridges always extending beyond caudal tergal margin on segments 2–19 (Fig. 9), spiniform caudally (Fig. 13) on segment 19 in both sexes or on segments 18 and 19 only in ♀. Axial line wanting to traceable in places. Transverse sulcus (Fig. 11) evident on segments 5–18 in ♂, likewise evident on segments 5–17, but only traceable on 18th in ♀, narrow, shallow, neither beaded at bottom nor reaching bases of paraterga. Stricture (Fig. 11) between pro- and metazona very faintly beaded at bottom dorsally. Pleurosternal carinae (Figs 10, 12) well-developed on segments 2-(9)10, traceable on (10)11–14(15), like low bosses on segments 15–17 in ♂, well-developed on segments 2-(9)10, visible on segments (10)11-(13)16, like low bosses on segments (14–16)17 in ♀, thereafter virtually absent in both sexes; with small caudal teeth (Fig. 10) on segments 3–5(9, 10) (♂) or 3(4)–5(7) (♀). Tergal setae fully abraded, pattern untraceable. Ozopores (Fig. 12) lateral, lying on callus ca. one-third metatergal length in front of caudal edge. Epiproct (Figs 13, 14) digitiform, flattened dorsoventrally, moderately long in lateral view, ratio of epiproct length to pre-epiproct length of telson 1: 2.2 in ♂ (Fig. 14); subtruncate and slightly concave to emarginate in dorsal view (Fig. 13); pre-apical papillae (Fig. 13) present, situated close to apex. Hypoproct (Fig. 15) roundly subtrapeziform caudally, 1+1 setae at caudal corners situated on well-separated knobs, sides straight to slightly concave.

Sterna moderately setose, not modified; a short, round, spiracle-bearing ridge flanking gonopod aperture (Fig. 16, arrows); each cross-impression with an evident transverse sulcus, but without axial groove. ♂ legs (Fig. 12) without tarsal brushes, ca. 1.5 times as long as midbody height, slightly shorter in ♀.

Gonopods (Figs 39–41, 45, 46, 53) peculiar in solenophore (**sph**) being rounded apically and considerably shorter than solenomere (**sl**), while its dentiform process (**dp**) more solid, and both **sph** and **dp** always placed behind solenomere in ventral view.

##### Remarks:

The sketch of a gonopod of *Chamberlinius shengmui* by Wang (1957) showed the solenophore being strongly broadened distad, while the solenomere broadened at midway. Apparently due to these distinctions, this species has since been considered as valid. We have been privileged to examine the specimens of *Chamberlinius shengmui* deposited at NMNH (without catalogue number) by Wang, and found the gonopod sketch of the solenomere accompanying the original description (Wang 1957) misleading. Having also re-examined the ♀ holotype and ♂ paratype of C. *piceofasciatus*, both housed in CAS, and compared them side-by-side with *shengmui*, we found that these species are identical. Therefore, *Chamberlinius shengmui* is a new junior subjective synonym of *Chamberlinius piceofasciatus*.

##### Distribution:

*Chamberlinius piceofasciatus* seems to be endemic to Taiwan, being restricted to higher elevations (2,000 to >3,000 m a.s.l) (Map).

#### Chamberlinius pessior sp. n.

urn:lsid:zoobank.org:act:F832D34E-4E24-4243-B39D-08A0B46E5B04

http://species-id.net/wiki/Chamberlinius_pessior

Figs 17–24 36–38 49–52

##### Holotype

 ♂ (NTNUL-My 74), Taiwan, Nantou County (南投縣), Renai Township (仁愛鄉), Lushan warm spring (廬山溫泉), ca. 1,200 m a.s.l., 29 August 1988, leg. S. H. Chen.

##### Paratypes:

 1 ♂, 5 ♀ (NTNUL-My 75*-*80), same locality and date, together with holotype.

##### Name:

 To emphasize the lower paraterga.

##### Diagnosis:

 Closest to *Chamberlinius hualienensis*, but differs in being obviously smaller, with paraterga like low ridges (versus higher ridges in *Chamberlinius hualienensis*), the pleurosternal carinae are with small caudal teeth on segments 3–10 (versus 3–7(8) in *Chamberlinius hualienensis*), legs with tarsal brushes (versus without in *Chamberlinius hualienensis*) and the gonopods showing the tip of the solenophore pointed and simple (versus bifid in *Chamberlinius hualienensis*).

##### Description:

**Figures 17–24.**
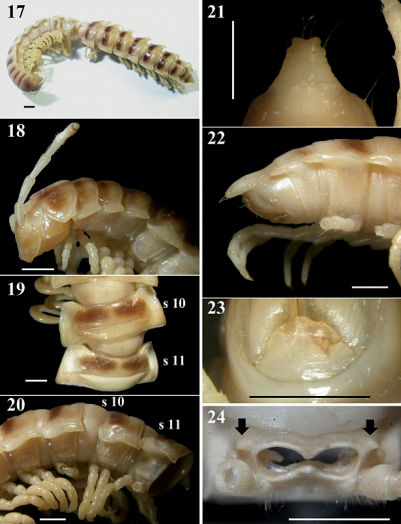
*Chamberlinius pessior* sp. n., holotype. **17** Entire body, dorsal view **18** Anterior body portion, lateral **19, 20** segments 10 and 11, dorsal and lateral views, respectively **21, 22** Epiproct, dorsal and lateral views, respectively **23** Hypoproct, ventral view **24** Spiracle-bearing cones lateral to gonopod aperture (arrows). Scale bar: 1.0 mm. **s10** and **s11**: segments 10 and 11 separately.

 Length 25–26 (♂, n = 2) or 27–29 mm (♀, n = 5), width of metazonite 10 ca. 3.5–3.8 (♂) or 3.8–4.0 mm (♀). Coloration in alcohol (Fig. 17) almost entirely light yellow-brown in ♂, but infuscate (brown) in ♀ compared with ♂; colour pattern same as in *Chamberlinius hualienensis*.

In width, head < collum = segment 2 > 3 < 4 << 5 < 6 < 7 < 8 < 9 < 10 < 11 < 12 < 13 < 14 < 15 <16 in ♂, or head < collum = segment 2 > 3 < 4 < 5 < 6 < 7 < 8 < 9 < 10 < 11–16 in ♀; thereafter body gradually and gently tapering both in width and height towards telson. Antennae (Figs 17, 18) long and slender, reaching behind middle of metatergum 4 (♂) or 3 (♀) dorsally. Surface generally shining and smooth, rugulose (**r**) (Figs 18, 20, 49) on metaterga on place and below paraterga 2–19, metazona below paraterga evidently and densely granular on segments 2–19 in both sexes. Paraterga (Fig. 19) very well-developed, calluses (Figs 19, 20) delimited by a sulcus dorsally and ventrally on segments 2-19; paraterga like low ridges (Figs 20, 49), slightly extending beyond caudal tergal margin on segments 2-4, obviously beyond it on segments 5-19 (Fig. 17), spiniform caudally (Fig. 22) on segments 17-19 in both sexes; anterior corner of paraterga thinner dorsoventrally and depressed only in ♂. Axial line traceable to evident on pro- and metaterga from collum to anterior part of segment 19 in ♂, fainter in ♀. Transverse sulcus (Figs 19, 49) evident on segments 5-17, traceable on segment 18, wanting on segment 19 in both sexes, narrow, shallow (markedly shallower in ♂ as compared to ♀), neither beaded at bottom nor reaching bases of paraterga. Limbus thin, caudal margin entire. Stricture (Fig. 19) between pro- and metazona roughly beaded, evidently more roughly so in ♀ as compared to ♂. Pleurosternal carinae (Figs 18, 20) well-developed on segments 2–10, traceable on segments 11–15 in ♂, well-developed on segments 2–12, visible on segments 13–17 in ♀, thereafter wanting, with small caudal teeth on segments 3–10 (♂) or 3–9 (♀). Tergal setae fully abraded, pattern untraceable in both sexes. Ozopores (Figs 20, 49) lateral, lying on callus about one-third metatergal length in front of caudal edge. Epiproct (Figs 21, 22) digitiform, long, flattened dorsoventrally, curved and directed caudoventrad in lateral view (Fig. 22), ratio of epiproct length to pre-epiproct length of telson 1: 2.0 in ♂ (Fig. 22), subtruncate and emarginated (♂) or slightly concave (♀) in dorsal view (Fig. 21); pre-apical papillae (Fig. 21) evident, situated close to apex. Hypoproct (Fig. 23) subtrapeziform, caudally convex, 1+1 setae at caudal corners situated on well-separated knobs, sides slightly concave in both sexes.

Sterna moderately setose in ♂, sparsely so in ♀; an obvious, short, round, spiracle-bearing ridge flanking gonopod aperture (Fig. 24, arrows); each cross-impression with an evident transverse sulcus, without axial groove. Legs (Fig. 49) with tarsal brushes, long, ca. 1.2 times midbody height in ♂, slightly shorter in ♀.

Gonopods (Figs 36–38, 50–52) as in *Chamberlinius hualienensis*, but solenophore (**sph**) not bifid, being simple and pointed.

##### Remarks.

*Chamberlinius pessior* sp. n. is endemic to Taiwan, being known from a single locality at 1,200 m a.s.l. (Map).

#### Chamberlinius sublaevus sp. n.

urn:lsid:zoobank.org:act:6AC5346F-0657-44A1-9452-59B2A1AC9B61

http://species-id.net/wiki/Chamberlinius_sublaevus

Figs 25–32 42–44, 47, 48 54–57

##### Holotype

 ♂ (NSYSUB-DI 50), Taiwan, Taichung County (台中縣), Heping Township (和平鄉), Syuan (思源), 1.5 km away from the entrance of forest path no. 710, ca. 2,050–2,100 m a.s.l., 21 August 2002, leg. C. C. Chen and Y. H. Lin.

##### Paratypes:

 3 ♂, 1 ♀, 2 juveniles (NSYSUB-DI 51-57), same locality and date as in holotype.1 ♂, 1 ♀ (NCHUL), Nantou County (南投縣), Renai Township (仁愛鄉), Meifeng (梅峰), ca. 2,000 m a.s.l., 2 April 2002, S. H. Wu. 1 ♂ (TFRI), Ilan County (宜蘭縣), Datong Township (大同鄉), Lakes Jialuohu (加羅湖), ca. 2,300 m a.s.l., 22 April 2001, leg. W. C. Yeh. 1 ♂ (TFRI), same place, 26 April 2002, same collector. 1 ♂ (TFRI), same place, 4 June 2003, same collector. 1 ♂, 1 ♀ (NSYSUB-DI 451-452), same township**,** Yuanping forest path (元平林道)**,** 4 km, on a dirty wall, ca. 1,990 m a.s.l., 29 August 2004, leg. H. D. Zhu.

##### Name:

 To emphasize the elevated paraterga.

##### Diagnosis:

Closest to *Chamberlinius piceofasciatus*, but differs in the smaller size and higher paraterga; by the pleurosternal carinae with small caudal teeth either on segments 3–8(10) (♂) or 3–4, or without any caudal teeth (♀) (versus segments 3–5(10) (♂) or 3(4)-5(7) (♀) in *Chamberlinius piceofasciatus*); and in gonopod structure, with the solenophore being slender, its tip pointed and placed ventrad under the solenomere (versus the solenophore being much stouter, blunt and remaining fully behind and above the solenomere in ventral view in *Chamberlinius piceofasciatus*).

##### Description:

**Figures 25–32.**
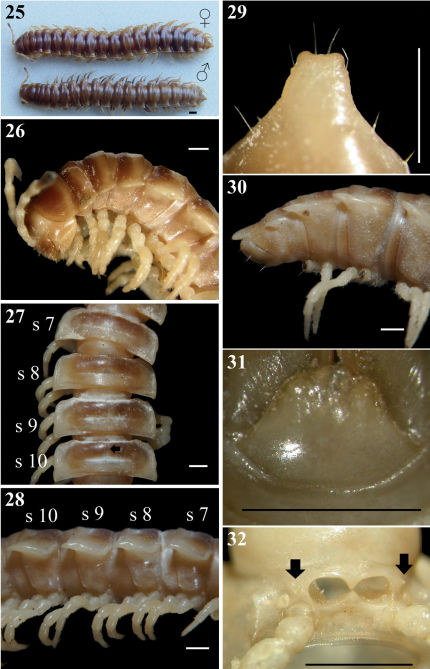
*Chamberlinius sublaevus* sp. n., ♀ and ♂ paratypes from Yuanping forest path (元平林道) (25–31)and Syuan (思源) (32), respectively. **25** Entire body, dorsal view **26** Anterior body portion, lateral **27, 28** Midbody segments, dorsal and lateral views, respectively. arrow: axial line **29, 30** Epiproct, dorsal and lateral views, respectively **31** Hypoproct, ventral view **32** Cones lateral to gonopod aperture (arrows). Scale bar: 1.0 mm. **s7-s10**: segments 7–10 separately.

 Length 29–30 (♂, n = 5) or 28 mm (♀, n = 1); width of metazonite 10 ca. 4–5 mm (♂) or 4–5.5 mm (♀). Coloration in alcohol (Fig. 25) pallid to light brown from head to end of epiproct, as well as from dorsum down to paraterga, sterna and legs; most of head (except for occipital part), anterior 2/3rds of collum (Fig. 27), and most of epiproct darker, colour pattern same as in *Chamberlinius piceofasciatus* (Gressitt, 1941) and similar in both sexes, but ♀ darker; antennae increasingly blackish distally, but tip contrastingly pallid.

In width, head <collum ≤ segment 2 > 3 = 4 << 5 < 6 < 7 < 8 ≤ 9 = 10 < 11–15 in ♂, or head < collum < segment 2> 3 = 4 << 5–16 in ♀; thereafter body gradually and gently tapering both in width and height towards telson. Antennae (Figs 25, 26) long, slender, reaching either middle to end of metatergum 4 dorsally in ♂, or end of metatergum 3 in ♀. Surface generally shining and rather smooth, sometimes rugulose on metaterga, rugulose, finely and densely granular below paraterga 2–19 (Figs 26–28, 55). Paraterga (Fig. 27) very well-developed, calluses delimited by a sulcus only dorsally on segments 3–4, both dorsally and ventrally on segments 5–19; paraterga like high ridges (Fig. 28) extending beyond caudal tergal margin on segments 5–19 (Fig. 25), spiniform caudally (Fig. 30) on segments 17–19 in both sexes. Axial line (Fig. 27, arrow) wanting to sometimes traceable (on prozona), visible also at anterior edge of collum to end of segment 19, or on segments 5–19, better so on ♂ metaterga than in ♀. Transverse sulcus (Figs 27, 55) evident on segments 5–18, wanting on segment 19 in ♂, present on segments 5–18(19) in ♀, narrow, shallow, neither beaded at bottom nor reaching bases of paraterga. Limbus thin, caudal margin entire. Stricture (Figs 27, 55) between pro- and metazona faintly beaded at bottom dorsally. Pleurosternal carinae (Figs 26, 28, 55) well-developed on segments 2–10, traceable on segments 11–13 in ♂, reduced to low bosses on segments 14–17 in ♂, well-developed on segments 2–8, visible on 9–16 (17) in ♀, thereafter virtually absent in both sexes; with small caudal teeth either on segments 3–8 or 3–10 (♂), or either on segments 3 and 4, or without any caudal teeth (♀). Tergal setae fully abraded, pattern untraceable. Ozopores (Figs 28, 55) lateral, lying on callus about one-third metatergal length in front of caudal edge. Epiproct (Fig. 29) digitiform, long, flattened dorsoventrally, ratio of epiproct length to pre-epiproct length of telson 1: 2.3 in ♂ (Fig. 30); subtruncate or emarginate in dorsal view; pre-apical papillae almost wanting, situated close to apex. Hypoproct (Fig. 31) subtrapeziform, caudally narrowly to broadly rounded, 1+1 setae at caudal corners situated on well-separated knobs, sides concave at base.

Sterna moderately setose, not modified; a pair of small, short, round, spiracle-bearing ridges flanking gonopod aperture (Fig. 32, arrows); each cross-impression with an evident transverse sulcus, without axial groove. ♂ legs (Figs 28, 55) evidently incrassate, especially so due to dorsally swollen prefemora, without tarsal brushes, ca. 1.7 times as long as midbody height, a little shorter and slenderer in ♀.

Gonopods (Figs 42–44, 47, 48, 54, 56, 57) very similar to those of *Chamberlinius piceofasciatus*, differing in solenophore (**sph**)being slender, elongate, pointed and directed ventrad under solenomere (**sl**).

##### Distibution:

 This new species is also endemic to Taiwan, being high-montane (2,000–2,300 m a.s.l.) (Map).

**Figures 33–38.**
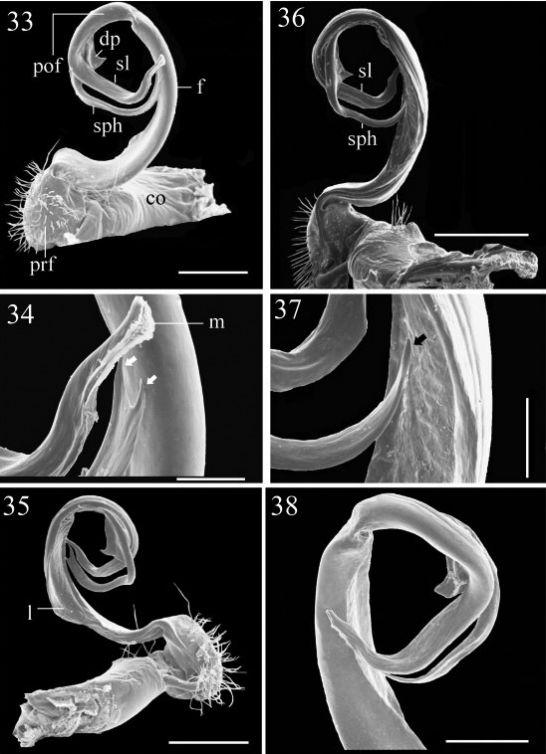
33–35 *Chamberlinius hualienensis* Wang, 1956, ♂ from CiiLan Forest Amusement Park (棲蘭森林遊樂園), left gonopod (33–35). **33** Entire gonopod, sublateral view **34** Tip of telopodite, sublateral view. Arrows: a bifid solenophore **35** Entire gonopod, medial view. **Figures 36–38.** *Chamberlinius pessior* sp. n., holotype, right gonopod. **36, 37** Entire gonopod and tip of solenophore (arrow), respectively, submedial view **38** Apical part of telopodite, medial and slightly ventral view. Scale bars = 0.5 mm (33, 35, 36); 0.1 mm (34, 37); 0.25 mm (38). **co**: coxa; **dp**: dentiform process; **f**: femorite; **m**: apical lamina; **l**: membranous lobe; **pof**: postfemoral region; **prf**: prefemur; **sl**: solenomere; **sph**: solenophore.

**Figures 39–44.**
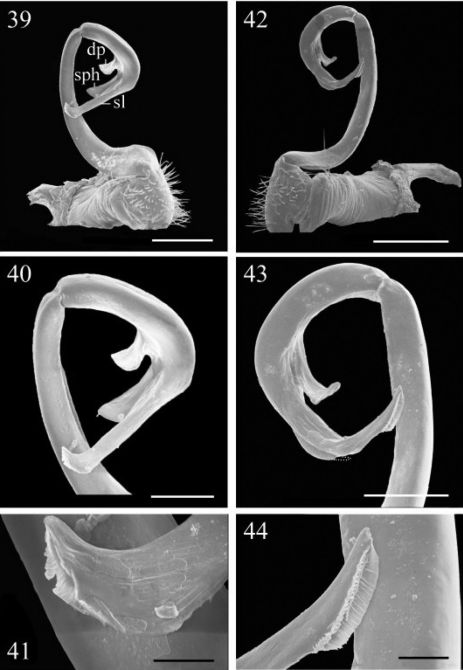
39–41 *Chamberlinius piceofasciatus* (Gressitt, 1941), ♂ from AliShan (阿里山), right gonopod, subventral view. **39** Entire gonopod **40** Apical part of telopodite **41** Tip of solenomere, respectively. **Figures 42–44.** *Chamberlinius sublaevus* sp. n.,♂ paratype from Syuan (思源), left gonopod, sublateral view. **42** Entire gonopod **43** Apical part of telopodite, dotted line shows a broken tip of solenophore **44 **Tip of solenomere. Scale bars: 0.5 mm (39, 42); 0.25 mm (40, 43); 0.05 mm (41, 44).

**Figures 45–46.**
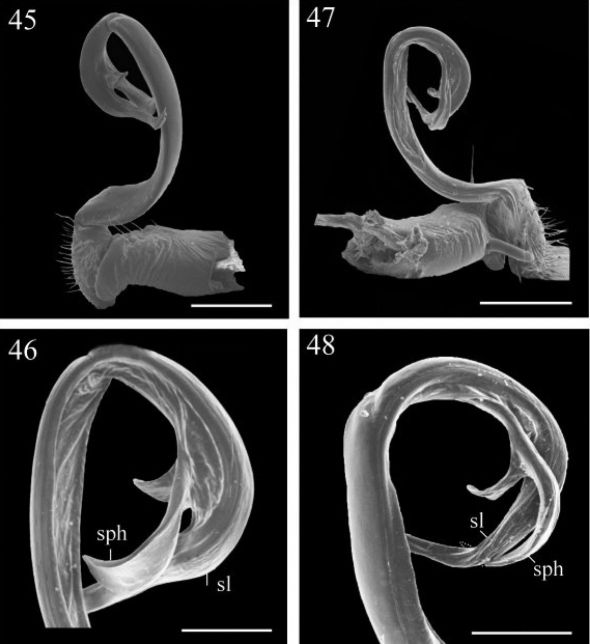
*Chamberlinius piceofasciatus* (Gressitt, 1941), ♂ from AliShan (阿里山), left gonopod, submedial view. **45** Entire, lateral view **46** Apical part of telopodite, submedial view. **Figures 47–48.** *Chamberlinius sublaevus* sp. n., ♂ paratype from Syuan (思源), left gonopod, submedial view. **47** Entire, dotted line shows a broken tip of solenophore **48** Apical part of telopodite. Scale bars: 0.5 mm (45, 47); 0.25 mm (46, 48). **sl**: solenomere; **sph**: solenophore.

**Figures 49–52.**
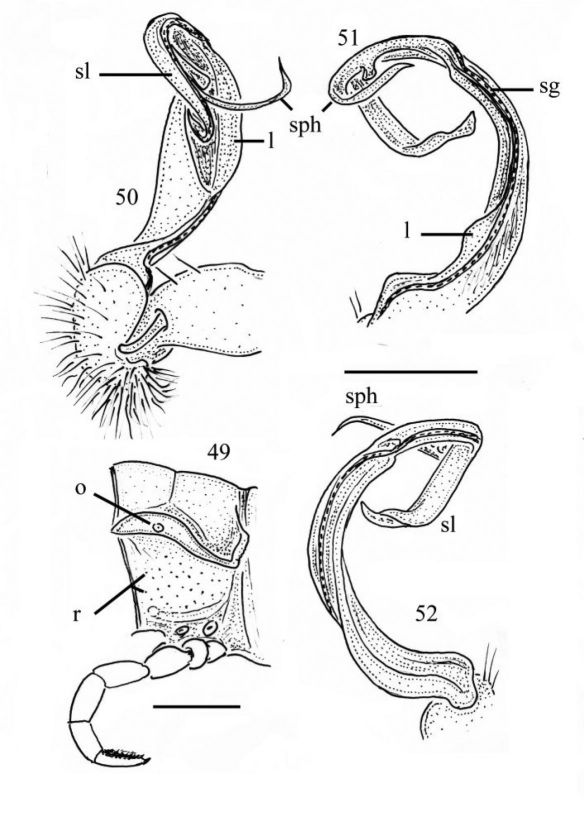
*Chamberlinius pessior* sp. n., holotype. **49** Segment 10, lateral view **50–52** Right gonopod, medial, dorsal and sublateral views, respectively. Scale bars: 1.0 mm (49); 0.5 mm (50–52). **l**: membranous lobe; **sg**: seminal groove ; **sl**: solenomere; **sph**: solenophore; **o**: ozopore; **r**: rugulosity.

**Figures 53–57.**
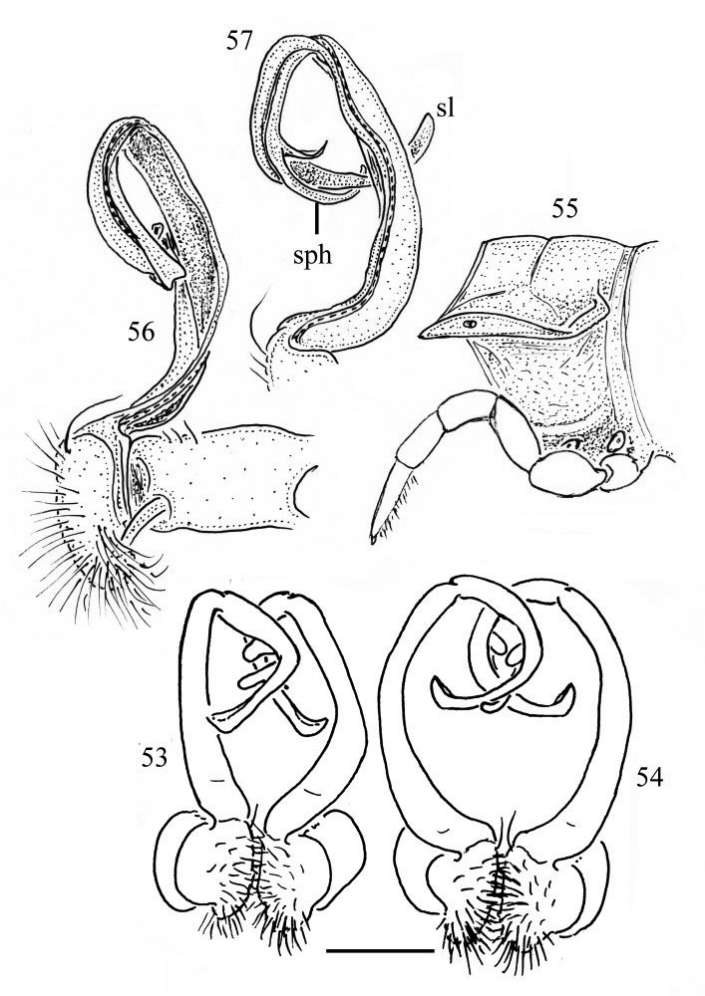
53 *Chamberlinius piceofasciatus* (Gressitt, 1941), ♂ from AliShan (阿里山), entire gonopods, ventral view. **Figures 54–57.** *Chamberlinius sublaevus* sp. n., ♂ paratypes from Meifeng (梅峰) (54) and Syuan (思源) (55–57). **54** Entire gonopods, ventral view **55** Segment 10, lateral view **56–57** Right gonopod, medial and dorsal views, respectively. Scale bars: 0.5 mm (53, 54); 1.0 mm (55–57).

## Key to Chamberlinius species (based mainly on adult males):

**Table:** 

1	Dark brown subtrapeziform markings on presulcus halves of metaterga 5–18 divided by a light axial line; solenophore as long as solenomere, parabasal dentiform process of gonopod membranous	2
–	Brown subtrapeziform markings on presulcus halves of metaterga 2–19 not divided by axial line; solenophore considerably shorter than solenomere, parabasal dentiform process of gonopod stout and more solid	3
2	Body much longer and wider (length 30–34 mm, width 4.5–5.0 mm); paraterga like high ridges; pleurosternal carinae with small caudal teeth on segments 3–7(8); solenophore with a bifid tip	*Chamberlinius hualienensis*
–	Body shorter and narrower (length 25–26 mm, width 3.5–3.8 mm); paraterga like low ridges; pleurosternal carinae with small caudal teeth on segments 3–10; solenophore with one pointed end	*Chamberlinius pessior* sp. n.
3	Larger (length 29–33 mm); paraterga like low ridges; parabasal dentiform process and solenophore of gonopods always placed behind solenomere in ventral view	*Chamberlinius piceofasciatus*
–	Smaller (length 29–30 mm); paraterga like high ridges; parabasal dentiform process placed behind solenomere, but solenophore pointed and directed ventrad under solenomere in ventral view	*Chamberlinius sublaevus* sp. n.

## Distribution

The distribution of *Chamberlinius* species in Taiwan shows that only one species, *Chamberlinius hualienensis*, is truly widespread across Taiwan, ranging from lowlands (ca 80 m a.s.l.) to high in the mountains (up to 2,500 m a.s.l.) (Map). Unsurprisingly, it is *Chamberlinius hualienensis* that seems to have become introduced to southern Japan. Another species, *Chamberlinius pessior* sp. n., has only been encountered at a single midmontane locality at 1,200 m a.s.l., while the remaining two congeners are far more local in distribution, being restricted to high elevations (> 2,000 a.s.l.) both in the northern and central parts of the island. Allopatry or parapatry are prevailing, but syntopic occurrences of two species, one of them usually *Chamberlinius hualienensis*, are not too rare, e.g. at Alishan, Hoping etc.

To our mind, it seems more logical to surmise that high-montane species in Taiwan tend to be more local in distribution, more inclined to endemism and less liable to introduction than those showing vaster distributions, including lowland habitats. So we are inclined to interpret the presence of the especially common and eurytopic *Chamberlinius hualienensis* in Japan as likely introduced from Taiwan.

## Reclassification of the Chamberlinini

The subfamily Chamberlininae, proposed by Wang (1957) for *Chamberlinius* alone, was originally diagnosed as showing a distally “toothed” seminal groove branch (= solenomere). Because this statement was incorrect, Hoffman (1973), who was the first to revise *Chamberlinius*, downgraded the Chamberlininae to the tribe Chamberlinini and reshaped it as comprising both *Chamberlinius* and *Riukiupeltis*. He reformulated the tribe’s diagnosis to emphasize that the terminal part of the gonopod lying distally of the postfemoral cingulum represents a single element with several subterminal branches, one of which carries the seminal groove. This diagnosis still holds valid and, based on several recent revisions, including the present one, now allows for another few East to Southeast Asian genera and species to be formally added to the tribe.

Thus, the genus *Haplogonosoma* Brölemann, 1916, with presumably two valid species (one ranging from central Honshu, Japan to Kurile Island, Russia, the other from Kyushu, Japan to ?Sumatra, Indonesia) shows highly elongate gonopods, in which the slender femorite is supplied with a distinct lateral sulcus. Distally of it, there is a cingulum demarcating a clear-cut geniculation followed by a very long, mesally directed, complex and strongly coiled postfemoral region. The latter is split from its base into a very long, flagelliform, fringed solenomere supported by an undivided, similarly long, slender, membranous lamina (= solenophore). The solenophore carries a pronounced basal outgrowth distodorsally.

Golovatch et al. (1995), who revised *Haplogonosoma*, referred this genus, albeit with hesitations, to the basically Papuan tribe Eustrongylosomatini. At present, however, we believe it is better placed in Chamberlinini.

*Aponedyopus* Verhoeff, 1939, with three species from Taiwan, shows moderately elongate gonopods, in which the femorite is enlarged parabasally, supplied by a distoventral outgrowth, but devoid of a sulcus before a clear-cut cingulum and geniculation. The latter is followed by a long, mesally directed, complex and moderately coiled postfemoral region split from its base into a very long, simple and flagelliform solenomere supported by an only subterminally biramous, similarly long, slender, membranous lamina (= solenophore). The solenophore carries an evident basal outgrowth distoventrally.

Chen et al. (2010), in their recent revision of *Aponedyopus*, have suggested transfer of this genus from the Tonkinosomatini to the Chamberlinini. This transfer is formalized below.

The genus *Geniculodesmus* Chen, Golovatch & Chang, 2008 has recently been erected (Chen et al. 2008) to accommodate a single species, *Geniculodesmus inexpectatus* (Attems, 1944), described from Mt Takao, Hachioujishi, Tokyo Prefecture, Japan and also recorded as likely an introduction at Kaohsiung, Taiwan. Its gonopods are elongate, the femorite is slender and devoid of both a distal outgrowth and a sulcus before a clear-cut cingulum and geniculation. The latter is followed by a long, mesally directed, rather simple and moderately coiled postfemoral region split from its base into a long, simple and flagelliform solenomere supported by an only subterminally bifid, similarly long, slender, membranous lamina (= solenophore). The solenophore carries a basal, caudally directed outgrowth.

*Geniculodesmus* has initially (Chen et al. 2008) been considered as being especially similar to both *Haplogonosoma* and *Aponedyopus*, so its formal assignment to Chamberlinini is likewise fully warranted.

As regards *Riukiupeltis* Verhoeff, 1939, its scope and diagnosis remain rather obscure. The type-species *Riukiupeltis yamashinai* Verhoeff, 1939, from the Ryukyus, Japan has been described as showing the gonofemorite long and slender, devoid of both a sulcus and a distal outgrowth before a clear-cut cingulum and geniculation. The postfemoral part is very long, slender, probably directed mesad, moderately coiled, devoid of any basal outgrowth, gradually attenuating towards a pointed tip. A solenomere is only depicted as likely broken off, branching off from the distal half of the solenophore (Verhoeff 1939).

**Figures 58–59.**
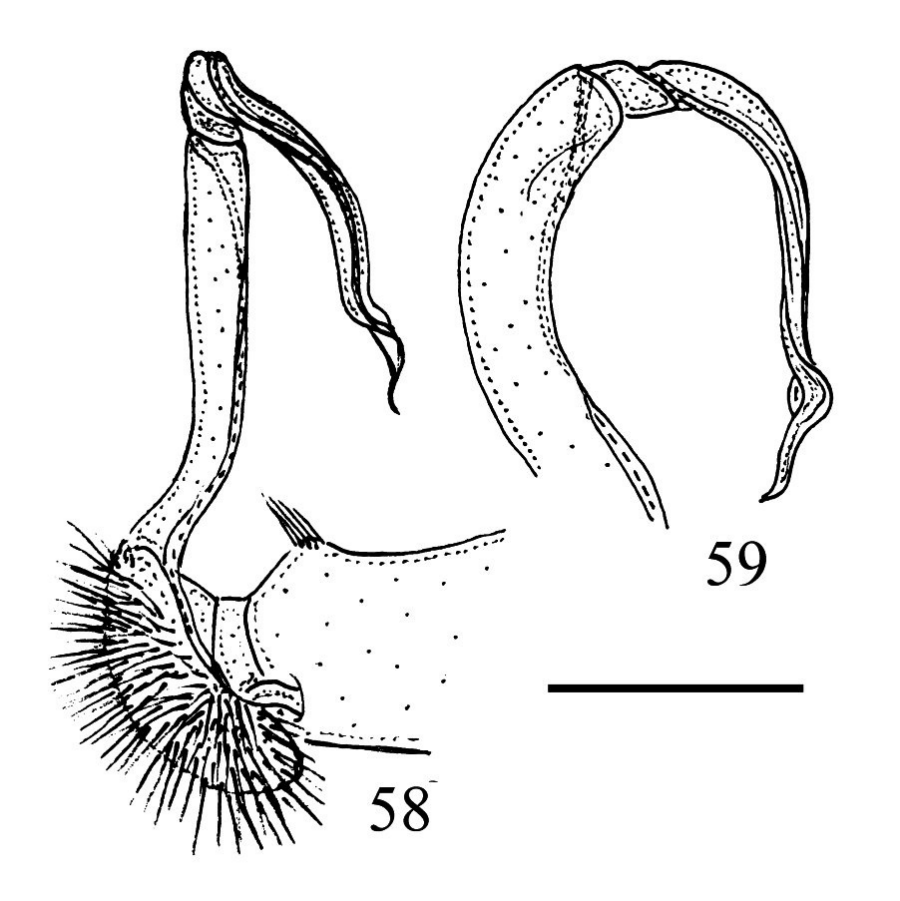
Right gonopod of *Riukiupeltis falcatus* (Attems, 1953), ♂ from Bi Doup National Park, southern Vietnam, medial and ventral views, respectively. Scale bar: 0.5 mm.

Such a conformation seemed so improbable that Jeekel (1968) doubted it altogether, especially as regards the extent and position of the solenomere. The more so as in another species, *Riukiupeltis falcatus* (Attems, 1953), from Laos and Vietnam, he also formally placed in *Riukiupeltis* the gonopods show a condition readily reminding of that observed in *Chamberlinius*: the solenomere is not flagelliform, but thick and long, gradually attenuating towards a pointed tip (Figs 58, 59). Yet, pending a revision of the type species *Riukiupeltis yamashinai*, it appears impossible to unequivocally state that *Riukiupeltis* is indeed characterized by the gonopod postfemoral portion being normal, non-split, devoid of any additional solenophore, being represented instead solely by a thick, long and helicoidally twisted solenomere.

To accommodate the above five genera into the tribe Chamberlinini, and to properly rediagnose it, only a few slight amendments to Hoffman’s (1973) definition are necessary, as follows.

### Tribe Chamberlinini

Wang, 1957

#### Diagnosis:

 Gonopod femorite usually long and slender, only rarely somewhat expanded parabasally and supplied with a distal outgrowth (*Aponedyopus*) or carrying a lateral sulcus (*Haplogonosoma*) before a clear-cut cingulum and geniculation. Postfemoral portion directed mesad, long, slender, more or less coiled, either entirely a thick solenomere devoid of any outgrowths (*Riukiupeltis*) or split at base into a thick (*Chamberlinius*) or thin, long, truly flagelliform solenomere and a membranous, more or less complex solenophore (remaining three genera).

#### Included genera:

*Chamberlinius* Wang, 1956, *Riukiupeltis* Verhoeff, 1939, *Aponedyopus* Verhoeff, 1939, *Haplogonosoma* Brölemann, 1916 and *Geniculodesmus* Chen, Golovatch & Chang, 2008.

## Supplementary Material

XML Treatment for Chamberlinius

XML Treatment for Chamberlinini
